# Dietary *Lactobacillus casei* K17 Improves Lipid Metabolism, Antioxidant Response, and Fillet Quality of *Micropterus salmoides*

**DOI:** 10.3390/ani11092564

**Published:** 2021-08-31

**Authors:** Jinsong Wang, Zhuoying Zhu, Shenghao Tian, Huiyu Fu, Xiangjun Leng, Lanming Chen

**Affiliations:** 1 College of Bioengineering, Jingchu University of Technology, 33 Xiang Shan Road, Jingmen 448000, China; jswang@jcut.edu.cn; 2 Laboratory of Quality and Safety Risk Assessment for Aquatic Products on Storage and Preservation (Shanghai), Ministry of Agriculture, College of Food Science and Technology, Shanghai Ocean University, Shanghai 201306, China; zhuzhuoying0410@163.com (Z.Z.); t1652138299@163.com (S.T.); fuhuiyu123@163.com (H.F.); 3 College of Fisheries and Life Science, Shanghai Ocean University, Shanghai 201306, China; xjleng@shou.edu.cn

**Keywords:** *Lactobacillus casei*, *Micropterus salmoides*, liver histology, antioxidant response, lipid metabolism, probiotics

## Abstract

**Simple Summary:**

In order to find effective antioxidants to improve the fleshy degeneration and liver tissue lesions of *Micropterus salmoides* that were fed artificial mixed feed, *Lactobacillus casei* K17, which displayed a high level of antioxidant activity in vitro and in vivo was selected in this study. The results indicated that after a trial for 69 days, live bacteria (LB), live bacteria protected by skim milk powder (MB), and dead bacteria were able to improve hemal and hepatic lipid metabolism and antioxidant response, reduce reactive oxygen species production, and protect *Micropterus salmoides* hepatic cells from injury, while LB and MB were also able to improve fillet quality. Therefore, *Lactobacillus casei* K17 might be a good alternative source of improving fillet quality and liver health in *Micropterus salmoides*.

**Abstract:**

We previously demonstrated that *Lactobacillus casei* K17, isolated from Korean kimchi, has high antioxidant levels in vitro and in vivo. However, its effect on *Micropterus salmoides* is unknown. In this study, we investigated the impact of *L. casei* K17 supplementation on the lipid metabolism, antioxidant response, liver histology, and fillet quality of *M. salmoides*. We randomly assigned 450 *M. salmoides* (33.0 ± 0.5 g) to six diet groups for 69 days. The diets were as follows: 0.85% normal saline; 10% skim milk powder; 1 × 10^8^ CFU/g live *L. casei* K17 (LB); 1 × 10^8^ live *L. casei* K17 protected by skim milk powder (MB); 1 × 10^8^ dead *L. casei* K17 (DB); and *L. casei* K17 fermentation supernatant. MB significantly improved the crude protein, total collagen, alkaline-insoluble collagen, fiber numbers, hardness, chewiness, and gumminess of *M. salmoides* fillets (*p* < 0.05). LB significantly improved crude protein and fiber numbers (*p* < 0.05). Furthermore, dietary supplementation with LB, MB, and DB maintained normal liver histology, preserved liver function, and increased hepatic and hemal antioxidant status by enhancing antioxidant enzyme activities. Meanwhile, the three diets also promoted lipid metabolism by increasing HDL-C effectiveness and reducing total cholesterol, triglyceride, and low-density lipoprotein cholesterol levels in serum and liver tissues, indicating that dietary supplementation with DB, LB, and MB had hypolipidemic effects on *M. salmoides*. MB and LB significantly improved fillet quality and LB, MB, and DB improved hemal and hepatic lipid metabolism and antioxidant response and reduced reactive oxygen species production, protecting *M. salmoides* hepatic cells from injury.

## 1. Introduction

*Micropterus salmoides* (largemouth bass) is an important carnivorous native of freshwater lakes and small rivers in North America [1]. It has been widely cultured in China because of its rapid growth, tender flesh, and high nutritional value and has become an economically valuable species of fish [2,3]. However, *M. salmoides* aquaculture in China mainly involves chilled or mixed fish feed, resulting in a waste of resources and environmental pollution. Furthermore, fleshy degeneration, liver tissue lesions, and infectious diseases were observed in *M. salmoides* that were fed artificial mixed feed, thus greatly restricting their intensive and large-scale breeding [4]. Oxidative stress may also induce hepatosis in fish [5,6]. Chen et al. reported that increased oxidative stress in *M. salmoides* that were fed oxidized lipids led to stimulated hepatic antioxidant defenses, vitamin E depletion in plasma and certain tissues, and pathological changes [5]. Yun et al. also reported that slight oxidation of these lipids in *M. salmoides* feed would negatively affect fish growth [6].

In commercial facilities, lipids in feedstuffs are readily susceptible to peroxidation, and heating or improper storage can also aggravate dietary lipid oxidation [5]. To maintain animal feed quality and prevent oxidative lesions in fish, effective antioxidants are needed in farming fish, such as endogenous free radical scavenging enzymes and exogenous antioxidants, such as vitamin C [7], vitamin E [8], and selenium [7,9]. *Lactobacilli*, which have been used as probiotics, show antioxidative functions, including reactive oxygen species (ROS) scavenging, metal ion chelation, enzyme inhibition, and reduction activity [10,11]. Rathore et al. reported that dietary supplementation of tilapia with 1 mg/kg of nano-Se could improve antioxidant activities (catalase; CAT), superoxide dismutase (SOD), glutathione peroxides, glutathione reductase, glutathione S-transferase, malondialdehyde (MDA), total antioxidant capacity (T-AOC), and disease resistance for 90 days. Xu et al. reported that dietary supplementation of juvenile tilapia (*Oreochromis niloticus*) with 100 IU Vitamin E and 10 g/kg alanyl–glutamine dipeptide in dried feed improved serum SOD, CAT, glutathione peroxidase (GSH-Px), and T-AOC in 18 °C freshwater for 12 weeks [8]. Dawood et al. reported that dietary supplementation of Nile tilapia (*Oreochromis niloticus*) with *L. plantarum* L-137 significantly increased SOD, CAT, and GSH-Px levels for 90 days (*p* < 0.05) [10]. In China, *L. casei*, *L*. *plantarum*, *Lactobacillus acidophilus*, and *Enterococcus faecalis* have been issued to be used as commercial feed additives [12].

Fish fillet quality is an important factor affecting consumer purchases and is substantially influenced by fish diet [13]. Recently, although attention has been increasingly paid to the quality of fish products, little is known about the effects of probiotics on the quality of fish fillets because the impact of probiotics on fish growth and immunity has been emphasized. In addition, Cao et al. reported that a 90-day dietary supplementation with 1 g/kg β-glucan and 1 × 10^9^ CFU/kg *B. cereus* significantly improved the fillet quality of Pengze crucian carp (*Carassius auratus*) in terms of hardness, springiness, cohesiveness, gumminess, chewiness, and resilience [14]. Yang et al. reported that dietary supplementation of carp (*Cyprinus carpio* L.) with 10^9^ CFU/kg *B. cereus* promoted antioxidant status, and significantly improved fish fillet texture, including hardness, gumminess, chewiness, and resilience, for 70 days [15].

In our previous studies, a number of *Lactobacilli* spp. were isolated from traditional Chinese fermented foods and identified [16]. We found that *L. casei* K17 displayed high level of antioxidant activity in vitro. In addition, live *L. casei* K17 cells significantly improved the growth performance and lipid and antioxidant levels in high-fat diet Wistar rats (Yang et al., unpublished). Furthermore, skim milk powder exhibited a more significant protective effect on *L. casei* K17 during both storage and consumption [17]. Although the probiotic potential of live *L. casei* as a dietary supplement in Channel catfish (*Ictalurus punctatus*) has been indicated [18], however, little is known about its effect on *M. salmoides*. *M. salmoides* is sensitive to dietary peroxidation [6]. For example, dietary supplementation of *M. salmoides* with 150–1500 mg/kg ethoxyquin effectively relieved oxidative stress induced by fish oil oxidation and alleviated hepatosis symptoms [6]. Therefore, the aim of this study was to investigate the effects of different forms of *L. casei* K17 on the antioxidant response, lipid metabolism, and fillet quality of *M. salmoides*. Given the heat treatment normally used in the aquaculture feed industry for extruded pellets normally used in fish farming, live and dead *L. casei* K17 as well as *L. casei* K17 protected by cryoprotectant skim milk powder were investigated to reduce possible loss of the probiotic strains in feed processing. The results of this study provide new insights into the preparation of antioxidant and anti-aging drugs, healthy products, and feed additives containing *L. casei* K17.

## 2. Materials and Methods

### 2.1. Experimental Diet and Culture Conditions of M. salmoides

The commercial feed for fish (Zhejiang Dongyu Biotechnology Co., Ltd., Huzhou, China) was used as a basic dietary source in this study. Six experimental diets and culture conditions of *M. salmoides* were prepared according to the methods described previously [19]. Briefly, live *L. casei* K17 cells were inoculated, harvested, and adjusted to 10^9^ CFU/mL cell slurry [19]. Fresh fermentation supernatant was collected by centrifuging the bacterium culture. Dead bacterial cells were obtained by heating the live cell slurry at 65 °C for 30 min. A 30 mL of the bacterial cell culture (Table 1) was sprayed into 300 g of the basic feed and then mixed, packed, stored at 4 °C, and used within 7 days. Then, 450 *M. salmoides* (33.0 ± 0.5 g) were randomly allotted to 18 cages (1.5 × 1.0 × 1.8 m) with 25 fish per cage and fed with six treatments diets by three replicates for 69 days. The feed in the SG group was used as a control. The fish were fed twice daily, 7 days per week, and the daily feed was approximately 4% of the average body weight of *M. salmoides*. Water temperature was 28–32 °C; dissolved oxygen was more than 4 mg/L; pH was 6.5–7.3 [19].

### 2.2. Sample Collection

At the end of the feeding trial, the fish in each cage underwent fasting for 24 h, and their numbers and body weights were calculated and measured. Based on the initial weight and final weight of the fish in different groups, the feeding ratios were calculated [19]. Three fish per cage were randomly collected and anesthetized using eugenol (Henan South Ranch Biotechnology Co., Ltd., Zhengzhou, China). Fish brain tissue was destroyed using an anatomical needle [20]. Blood samples were collected from the randomly chosen fish through the caudal vein using a 1 mL syringe. The samples were immediately transferred into sterile 2 mL Eppendorf tubes without an anticoagulant and then allowed to clot for 1 h at 25 °C and for 4 h at 4 °C. Serum was obtained by centrifugation of the clotted samples at 1500× *g* for 5 min at 4 °C. Fish abdomens were cleaned with 75% ethanol, and the abdominal cavity was opened under aseptic conditions. Fish livers were collected and divided into two parts: the part of the liver tissue (0.5 × 0.5 × 0.5 cm) near the bile duct was immersed in a sterile 5 mL Eppendorf tube with 4% polysorbate solution for histological analysis, and the remaining tissue was collected into a sterile 5 mL Eppendorf tube and stored at −80 °C until assayed. The serum and liver samples of the randomly chosen fish were then subjected to T-AOC, SOD, MDA, GSH-Px, total cholesterol (TC), triglyceride (TG), low-density lipoprotein cholesterol (LDL-C), and high-density lipoprotein cholesterol (HDL-C) assays. Fillet samples from the dorsal sides of the fish (Figure S1) were collected for further composition and quality analysis.

### 2.3. Determination of Fillet Water, Crude Protein, Crude Fat, Crude Ash, and Collagen of Fish Samples

All chemical analyses of the fillets were conducted according to the methods of the Association of Official Analytical Collaboration (AOAC) International (2006). Three fish fillets per group were placed in an oven to measure moisture. The subsequent dried fish samples were ground into powders. A 2 g sample of the powder was then subjected to a muffle furnace (SXL-1216, Shanghai Jinghong Experimental Equipment Co., Ltd., Shanghai, China) at 550 °C for 16 h to measure ash. Another 1 g was used to measure crude lipids using *Tecator* *Soxtec* *System* *HT* *1043* Extraction Unit (Foss, Hillerød, Denmark), and a 0.2 g sample was used to measure crude protein using the Kjeldahl method in a Kjeldahl nitrogen analyzer (Kjeltec 2300 Analyzer, Foss Tecator, Sweden) [21].

The method outlined by Zhang et al. [22] was used to determine alkaline-soluble and alkaline-insoluble hydroxyproline (Hyp) in fillets. A mixture of 1 g fish fillet sample and 9 mL cold distilled water was homogenized at 4 °C for 1 min and then agitated at 4 °C for 4 h on a shaker after mixing 10 mL 0.2 mol/L cold sodium hydroxide with it. It was then centrifuged at 10,000× *g* at 4 °C for 30 min, and the supernatant and sediment were carefully collected for alkaline-soluble and alkaline-insoluble collagen analysis, respectively. The total Hyp content was measured using kits from Nanjing Jiancheng Bioengineering Institute (Nanjing, China). An ultraviolet spectrophotometer (UV759, Shanghai Precision Scientific Instrument, Shanghai, China) was used to measure Hyp content at a wavelength of 560 nm. Collagen content was calculated by multiplying the Hyp content by eight, according to the AOAC method 990.26 (AOAC 2000), which indicates that Hyp content in collagen is 12.5% if the nitrogen–to–protein factor is 6.25.

### 2.4. Quality Analysis of Fish Fillets

One piece of the left fillets (1 × 1 × 1 cm) from the dorsal side were obtained from three fish per cage (Figure S1B) and measured within 2 h using a Universal TA texture analyzer equipped with a cylindrical probe (36 mm diameter) (Shanghai Tengba Instrument Technology Co., Ltd., Shanghai, China). Nine fish were tested for each of the six treatment groups. Hardness, stickiness, springiness, chewiness, gumminess, cohesiveness, and resilience were determined based on a method developed by Cao et al. [15]. The compression rate was set to a test speed of 1 mm/s, and the deformation was 60% of fillet thickness. The samples were compressed twice, 60 s apart (*n* = 9).

### 2.5. Liver and Fillet Histology

Based on the method described by Torrecillas et al. and Xu et al. [23,24], one liver tissue (0.5 × 0.5 × 0.5 cm) near the bile duct and one part (0.5 × 0.5 × 0.5 cm) of the dorsal fillets per cage were immersed in 4% polysorbate solution for 24 h. These were then dehydrated in a series of alcohol solutions and embedded in paraffin. The sections (5 μm) were stained with Mayer’s hematoxylin and eosin (Wuhan Servicebio Co., Ltd., Wuhan, China). The morphological structures of both the fillet and the liver tissue were observed using an imaging microscope (×40, Nikon YS100, Tokyo, Japan). The fillet and liver tissue were quantified in four randomly selected visual fields in triplicate in each treatment. Fillet fibers were measured using image analysis software (Image14) [25]. Each image represented an area of 0.59 mm^2^. All fibers in the area were counted, excluding those in contact with the right and bottom edges.

### 2.6. Determination of Antioxidant and Lipid Indexes in Liver and Blood

According to the method described by Tan et al. [26], 2 mL serum and 2 g liver were collected, stored in dry ice, and sent to Nanjing Jiancheng Biological Co., Ltd. (Nanjing, China); the corresponding kits were used to determine total bile acid (TBA), T-AOC, SOD, MDA, GSH-Px, TC, TG, LDL-C, and HDL-C in both types of samples.

### 2.7. Statistical Analysis

All determinations were performed in triplicate, and all results are presented as mean ± standard error (SE) or mean ± standard deviation (SD). Statistical analyses were performed using SPSS version 22.0. The texture parameters of group differences were studied with covariance analyses using the final weight values as the covariate and other data were analyzed using one-way analysis of variance and post-hoc Duncan multiple range tests [27]. A significant difference was defined at *p* < 0.05.

## 3. Results

### 3.1. Fillet Moisture, Crude Protein, Crude Fat, Crude Ash, and Collagen of M. salmoides

**Figure 1:**
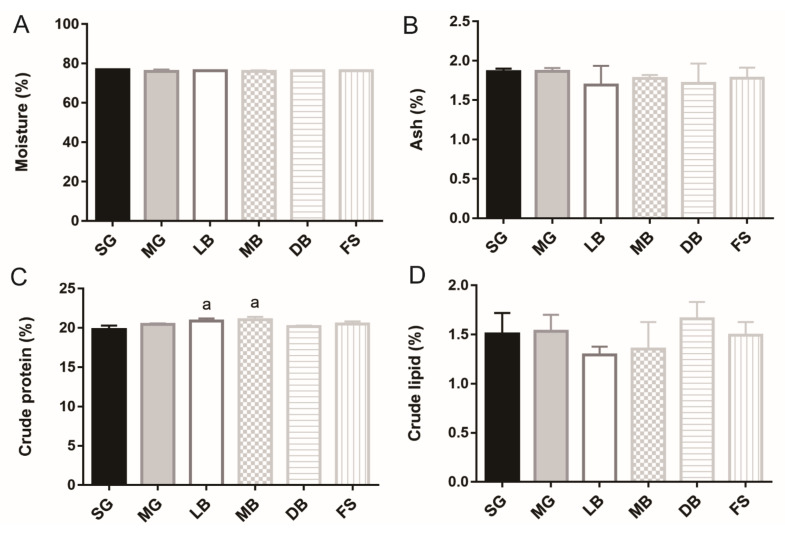

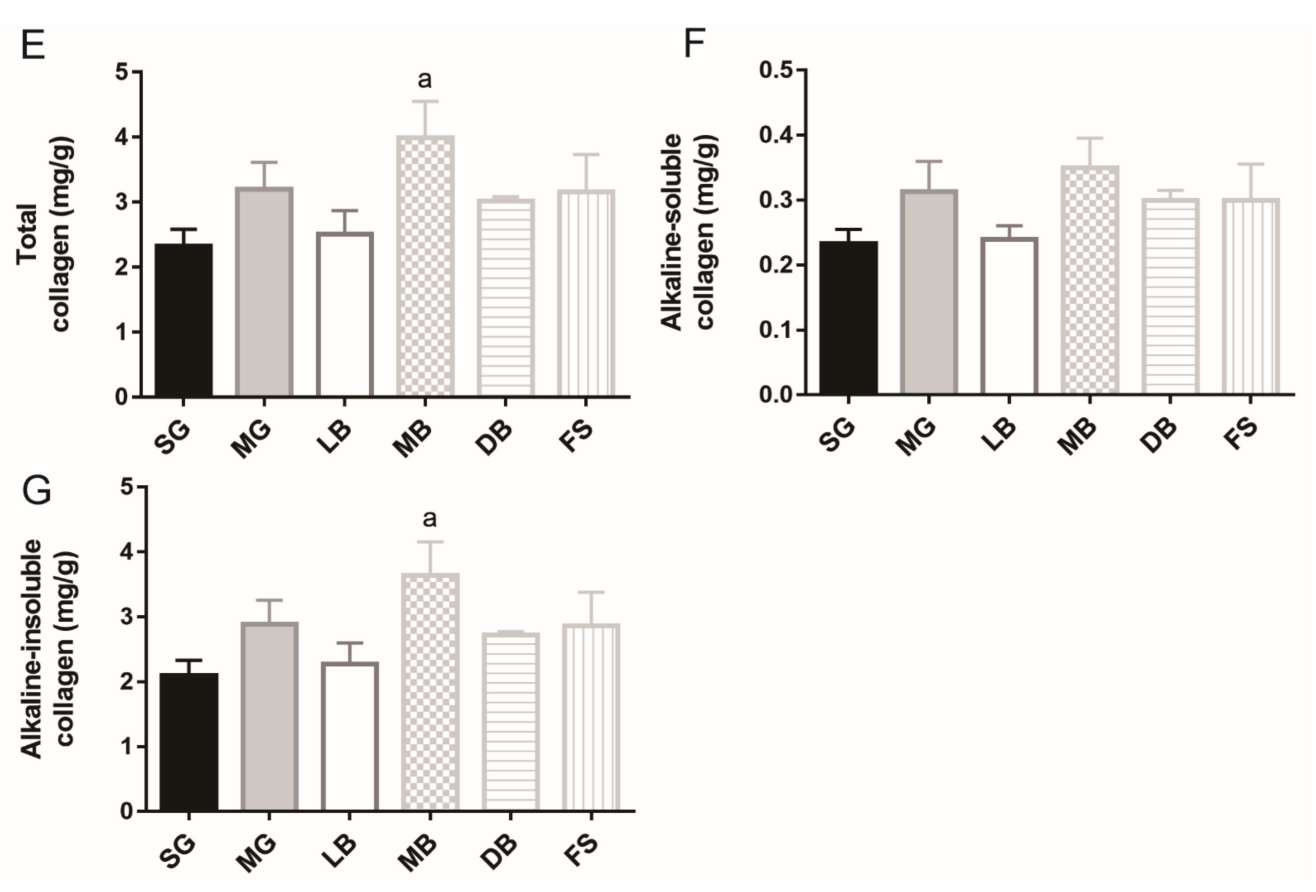
Effects of dietary supplementation with *L. casei* K17 on *M. salmoides* fillet composition. (**A**) Moisture. (**B**) Ash. (**C**) Crude protein. (**D**) Crude lipid. (**E**) Total collagen. (**F**) Alkaline-soluble collagen. (**G**) Alkaline-insoluble collagen. Values (mean ± SE, *n* = 9), ^a^ significantly different from SG, *p* < 0.05.

The fillet compositions of *M. salmoides* in different treatment groups are illustrated in Figure 1. No significant difference in the moisture, ash, or crude lipid was observed in the MG, LB, MB, DB, and FS treatment groups when compared with the SG group (Figure 1A,B,D). The crude protein contents of the fish in the LB and MB groups were statistically different (1.1 times higher) from that in the SG group (*p* < 0.05) (Figure 1C). The total collagen and alkaline-insoluble collagen contents of the fish in the MB group were statistically different (2.4, l.7, and 1.7 times higher, respectively) from those in the SG group (*p* < 0.05) (Figure 1E,G). Thus, MB was found to increase the contents of crude protein, total collagen, and alkaline-insoluble collagen, while LB only increased crude protein content.

### 3.2. Fillet Histology of M. salmoides

**Figure 2:**
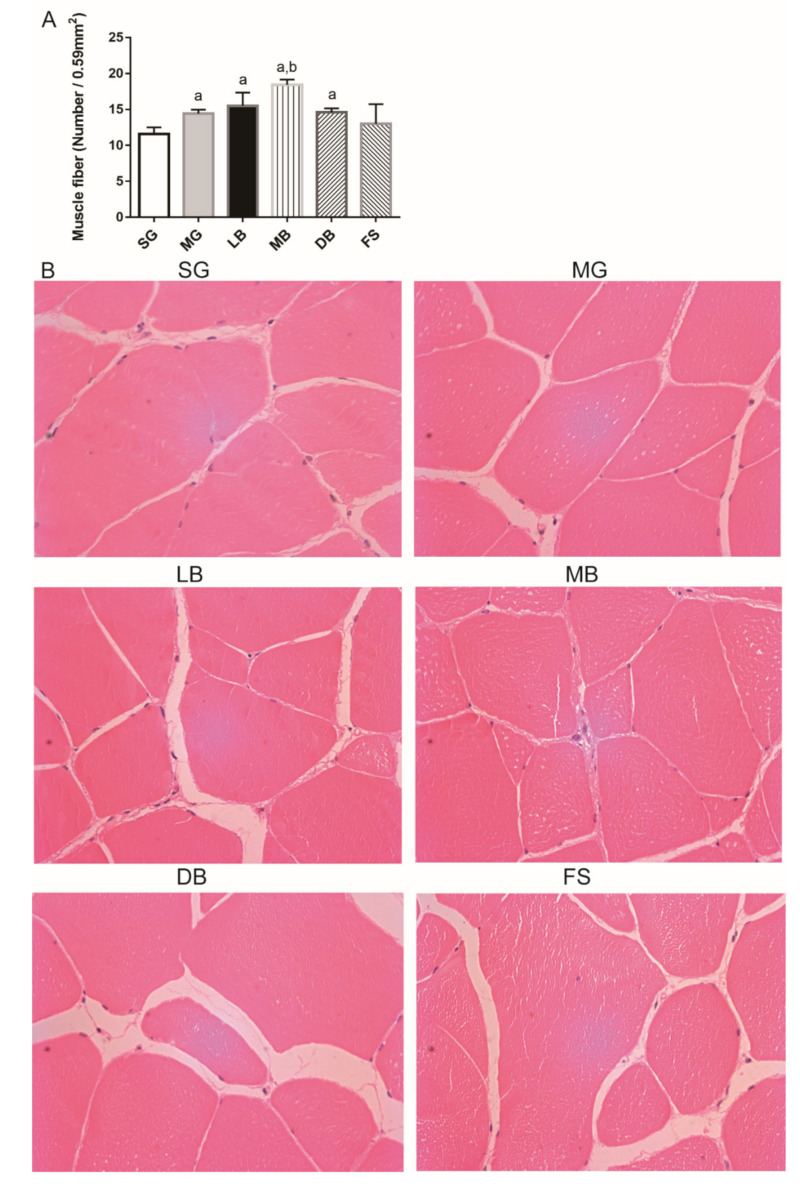
Effects of dietary supplementation with *L. casei* K17 on *M. salmoides* fillet fibers. (**A**) Average number value of fillet fiber according to fillet unit area. (**B**) Microscopic observation of fillet tissue (×40). Values (mean ± SE, *n* = 9), ^a^ significantly different from SG, ^b^ MB significantly different from MG, *p <* 0.05.

As shown in Figure 2A, the fillet fiber numbers of *M. salmoides* in the MG, LB, MB, and DB groups were significantly higher (1.2, 1.3, 1.6, and 1.3 times, respectively) than those in the SG group (*p* < 0.05). Meanwhile, fillet fiber numbers of the fish in the MB group were significantly higher (1.3 times) than those in the MG group (*p* < 0.05) (Figure 2A,B). Therefore, the MG, LB, MB, and DB diets were found to increase fillet fiber numbers; the MB diet was the most effective in improving this parameter.

### 3.3. Fillet Texture

The association between fillet texture and final weight of *M. salmoides* was analyzed (Table 2). Before adjustment, the hardness, chewiness, and gumminess of the fish in the MB group were significantly higher than those in the SG group (*p* < 0.05). No significant differences were observed in the stickiness, springiness, and cohesiveness of the fish in the MG, LB, MB, DB, and FS treatment groups when compared with those in the SG group. It is generally known that the fillet texture is usually influenced by individual weight of the fish. After adjustment for final weight, only the chewiness of the fish in the MB group was significantly higher than in the SG group (*p* < 0.05). These results indicated that MB improved fillet texture by increasing chewiness.

### 3.4. Liver Histology

**Figure 3:**
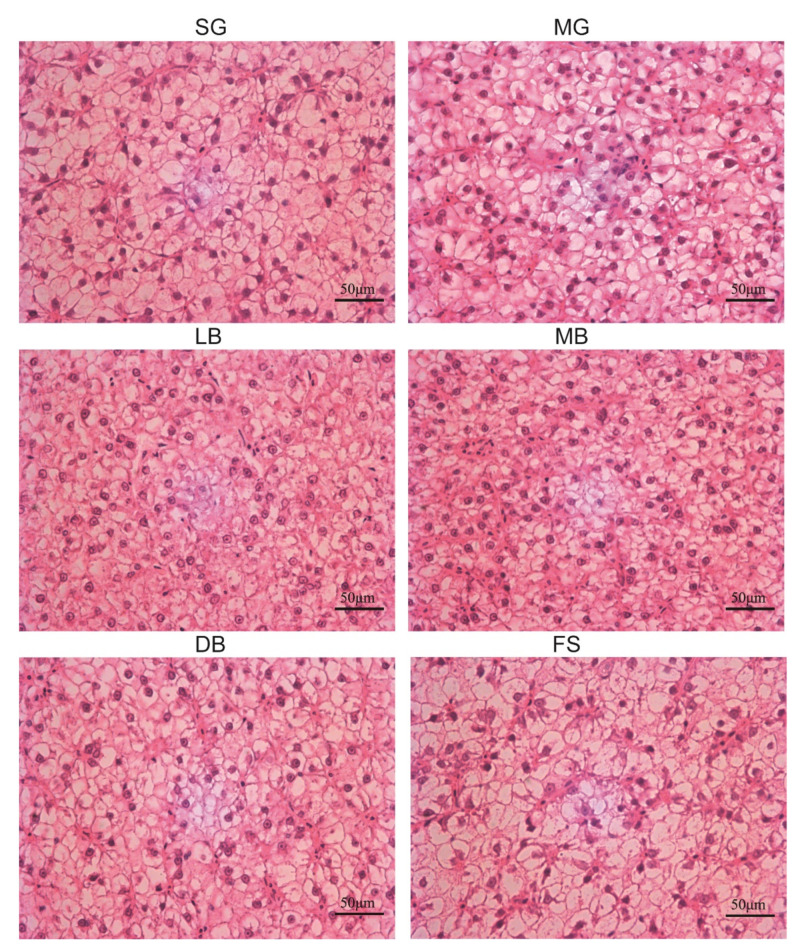
Liver cells of *M. salmoides* fed with six experimental diets of *L. casei* K17 (×40).

As shown in Figure 3, liver tissue showed cell infiltration and most cells showed cell enlargement in the SG group. In addition, cell vacuolization, nucleus deviation, cell membrane dissolution, and other phenomena were also observed to be serious. In the MG group, the degree of the five above mentioned phenomena was reduced to a certain extent as compared to the SG group. In the LB group, these cell characterization phenomena were further improved and restored. In the MB group, phenomena such as cell enlargement and vacuolization were greatly improved to the extent that they resembled normal cells. In the DB group, although the morphological observation of liver cells showed the existence of the abovementioned abnormal indicators, the degree of abnormality was obviously reduced. The liver cell morphology in the FS group was almost unchanged.

### 3.5. Antioxidant Response

**Figure 4:**
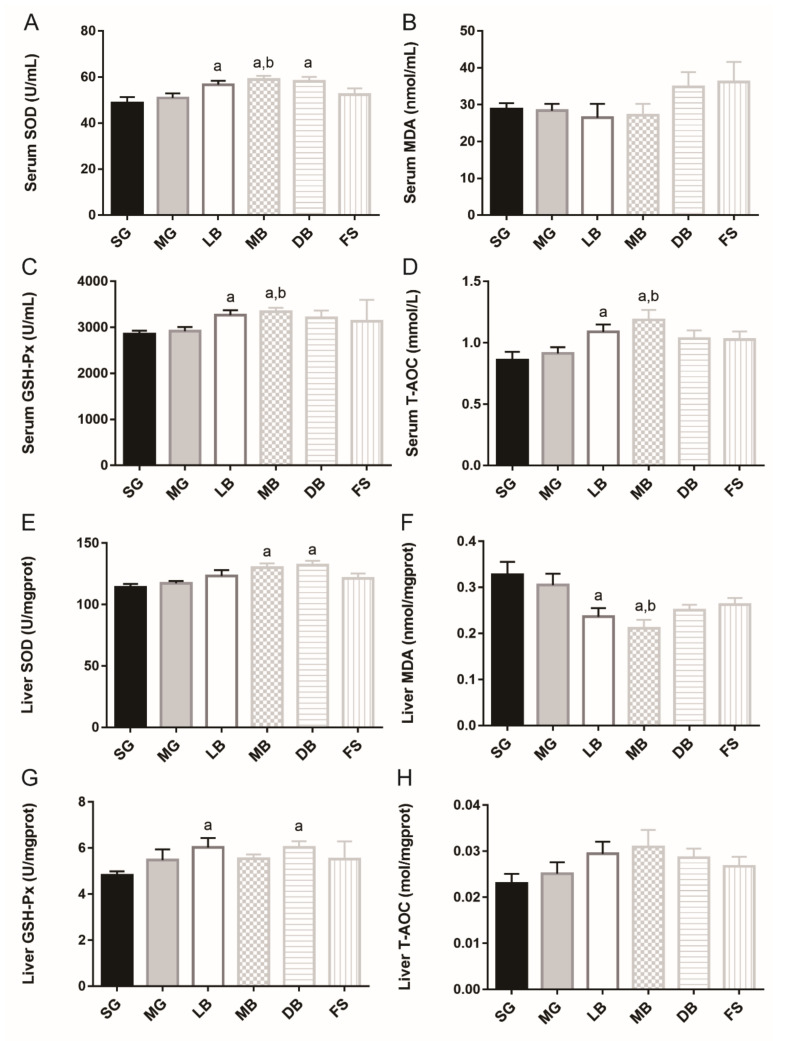
Effects of dietary supplementation with *L. casei* K17 on antioxidant capacity in *M. salmoides* serum and liver. Values (mean ± SE, *n* = 9), ^a^ significantly different from SG, ^b^ MB significantly different from MG, *p* < 0.05. (**A**) Serum SOD. (**B**) Serum MDA. (**C**) Serum GSH-Px. (**D**) Serum T-AOC. (**E**) Liver SOD. (**F**) Liver MDA. (**G**) Liver GSH-Px. (**H**) Liver T-AOC.

In the serum, compared to SG, LB, MB, and DB significantly and equally enhanced the SOD activity of *M. salmoides* (1.2 times) (Figure 4A). LB and MB significantly and equally increased GSH-Px activity (1.2 times) (Figure 4C), and LB and MB significantly increased T-AOC activity (1.3 and 1.4 times, respectively) (Figure 4D, *p* < 0.05). Meanwhile, MB significantly enhanced SOD activity 1.2 times and MB improved GSH-Px activity and T-AOC activity (1.1 and 1.3 times, respectively), compared with MG, (Figure 4A,C, *p* < 0.05). However, no significant differences were observed in serum MDA activities among the treatments (Figure 4B, *p* > 0.05). In liver tissue, as compared with SG, MB and DB significantly elevated SOD activity (1.1 and 1.2 times, respectively) (Figure 4E); LB and MB significantly decreased MDA activity (1.3 and 1.4 times, respectively) (Figure 4F), and LB and DB significantly and equally increased GSH-Px activity (1.3 times) (Figure 4G, *p* < 0.05). Meanwhile, MB significantly decreased MDA activity by 1.4 times (Figure 4F, *p* < 0.05).

### 3.6. Lipid Metabolism of M. salmoides

**Figure 5:**
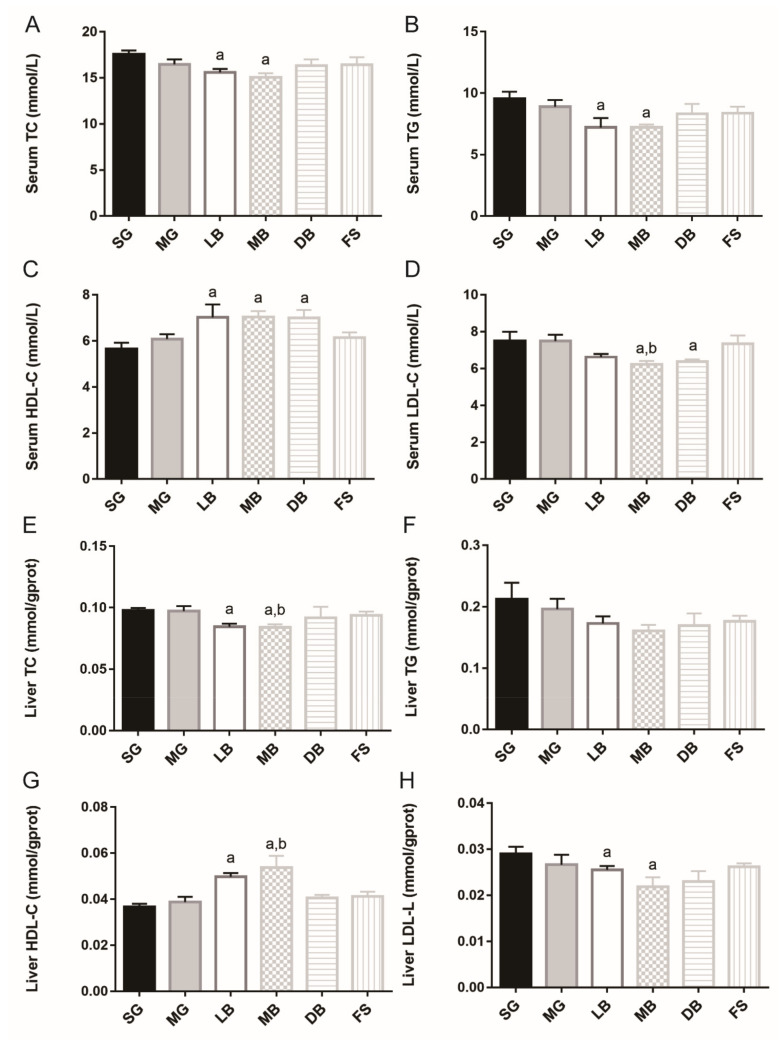
Effects of dietary supplementation with *L. casei* K17 on serum and liver lipids of *M. salmoides*. Values (mean ± SE, *n* = 9), ^a^ significantly different from SG, ^b^ MB significantly different from MG, *p <* 0.05. (**A**) Serum TC. (**B**) Serum TG. (**C**) Serum HDL-c. (**D**) Serum LDL-c. (**E**) Liver TC. (**F**) Liver TG. (**G**) Liver HDL-c. (**H**) Liver LDL-c.

**Figure 6:**
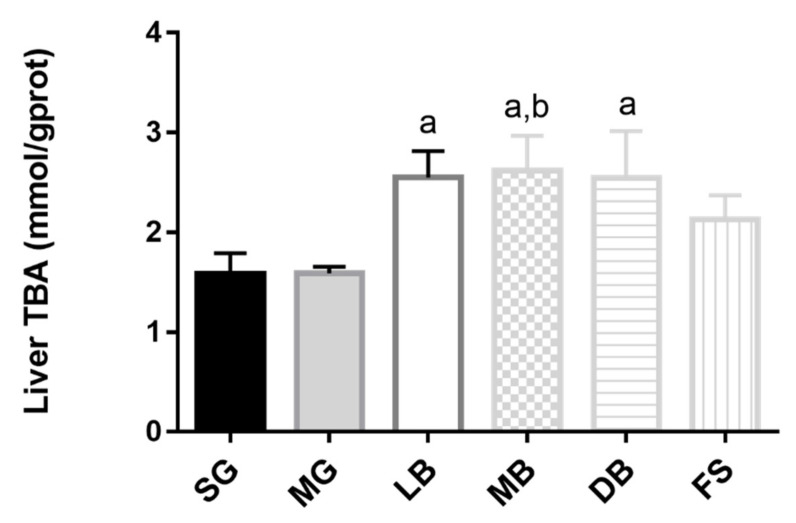
Effects of dietary supplementation with *L. casei* K17 on liver TBA of *M. salmoides*. Values (mean ± SE, *n* = 9), ^a^ significantly different from SG, ^b^ MB significantly different from MG, *p* < 0.05.

In serum, as compared to SG, MB and LB significantly decreased TC activity (1.2 times and 1.1 times, respectively) (Figure 5A) and equally decreased TG activity of *M. salmoides* (1.3 times) (Figure 5B). LB, MB, and DB significantly increased HDL-C activity (1.2, 1.3, and 1.2 times, respectively) (Figure 5C), and MB and DB significantly and equally decreased LDL-C activity (1.2 times) (Figure 5D, *p* < 0.05). Meanwhile, MB significantly decreased LDL-C activity by 1.2 times compared to MG (Figure 5D, *p* < 0.05). In liver tissue, compared with SG, LB and MB significantly and equally decreased TC activity (1.2 times) (Figure 5E), and increased HDL-C activity (1.4 and 1.5 times, respectively) (Figure 5G). MB and DB significantly and equally decreased LDL-C activity (1.3 times) (Figure 5H, *p* < 0.05). Meanwhile, MB significantly decreased TC activity by 1.2 times (Figure 5E) and increased HDL-C activity by 1.2 times (Figure 5G), compared with MG. However, no significant differences were observed in liver TG activities (Figure 5F, *p* > 0.05). Additionally, liver TBA content in the LB, MB, and DB groups was significantly increased than that in the SG group, and the liver TBA content in the MB group was significantly higher than that in the MG group (Figure 6, *p* < 0.05).

## 4. Discussion

Product quality is one of the most important factors affecting consumers’ purchase of fish products. Previous studies reported that dietary administration of *Bacillus* had no significant differences in the moisture, crude ash, crude lipid, and crude protein contents of Indian major carp (*Cirrhinus mrigala*) [28], Japanese flounder (*Paralichthys olivaceus*) [29], and Pengze crucian carp (*Carassius auratus var. Pengze*) [14]. Other studies have indicated that probiotics in fish diet can increase protein content but reduce lipid content of Asian Sea Bass (*Lates calcarifer*), Rainbow Trout (*Oncorhynchus mykiss*), and Javanese carp (*Puntius gonionotus*, Bleeker 1850) [30,31,32]. Meanwhile, Rodrigez et al. reported elevated lipid levels in juvenile Senegalese sole (*Solea senegalensis*, Kaup 1858) after probiotic feeding [33]. In this study, based on the rearing conditions, numbers of samples, and age of *M. salmoides* in the experiments, the fish density (about 1 kg/mc) was much lower than that of commercial farming (about 15 kg/mc), which may affect the efficacy of feed used. However, the MB diet was found to significantly increase crude protein, and collagen levels in *M. salmoides* fillets. The LB diet, on the other hand, significantly increased crude protein content, but no significant differences were observed in ash and moisture among the treatments. A higher body protein content in the LB and MB groups implies that by supplementing live *L. casei* K17 for 69 days, *M. salmoides* could more effectively convert the ingested feed into structural protein, which subsequently resulted in maximum muscle growth, a desirable aspect in fish farming. As the major component of connective tissues, collagen influences the functional and textural properties of fish fillets, and it is also the main contributor to the tensile strength of fish flesh [34]. Therefore, a high collagen content enhances fillet firmness [35]; for example, the increased flesh firmness of Atlantic salmon (*Salmo salar* L.) was mainly attributed to its high collagen content [36]. Collagen comprises alkaline-soluble and alkaline-insoluble collagen [37]. Alkaline-soluble collagen includes degraded collagen and newly synthesized collagen molecules, while alkaline-insoluble collagen includes intact, mature, and crosslinked collagen molecules, forming a resilient network, and making a significant contribution to the tensile strength of fish fillets. Sun et al. reported that dietary supplementation of 600–1000 mg/kg geniposidic acid or 20 g/kg *Eucommia ulmoides* increased total collagen and alkaline-insoluble collagen content in grass carp (*Ctenopharyngodon idella*) in a 75-day feeding trial [38]. The present study showed that dietary MB significantly increased total collagen content, which comprised alkaline-insoluble collagen, in *M. salmoides* fillets. Pyridinoline crosslinks mainly exist in alkaline-insoluble collagen, and it is an important substance characterizing flesh quality [37]. Further research is required to examine whether MB influences collagen maturity and pyridinoline formation.

In addition to nutrient composition, fillet quality also includes texture in terms of hardness, springiness, chewiness, cohesiveness, and stickiness, all of which are widely used to evaluate fish meat quality. Previous studies have indicated that dietary supplementation with *L. plantarum* and *B. cereus*, and a mixture of β-glucan and *B. subtilis*, can improve the hardness, stickiness, chewiness, gumminess, and resilience of Pengze crucian carp (*Carassius auratus*) [15]. In this study, the MB diet was found to improve fillet texture by increasing chewiness.

Flesh quality is closely related to the number and size of fillet fibers [39]. Fillet growth was directly correlated with the density and diameter of fillet fibers, which can be modified through nutrition [40]. In mammals, the number of fillet fibers does not change after birth [41], but it continues to increase throughout the life cycle of several fish [42]. Jhonston et al. reported that fillet fiber density was positively and linearly correlated with meat quality, “chewiness”, which had the most significant correlation, “firmness”, “mouth-feel”, and “dryness” [43]. In this study, the MG, LB, MB, and DB diets increased fillet fibers compared to the SG diet (*p* < 0.05). Therefore, all four types of diets were beneficial for improving *M. salmoides* fillet quality.

Excessive ROS production in animals leads to a deterioration in meat quality, including its nutritional and sensory quality [44,45]. Aberle et al. reported that ROS affected the turnover of collagen and meat tenderness in bovine fillets because it increased the activity of matrix metalloproteinase-2, which was responsible for collagen degradation through intramuscular fibroblasts [46]. In this study, the addition of MB improved the antioxidant defense mechanism, which led to reduced ROS generation and increased total collagen and alkaline-insoluble collagen. This consequently led to an increase in meat tenderness. Accordingly, *L. casei* K17 may partly influence meat quality by elevating the antioxidant status of *M. salmoides*.

The antioxidant capacity of a body’s defense system is closely related to health. SOD and GSH-Px are important antioxidant enzymes, and T-AOC directly reflects the antioxidant capacity of fish. MDA levels, on the other hand, indirectly reflect the severity of damage to fish body cells after a free radical attack [47,48,49]. For example, Zhang et al. reported that *Cyprinus carpio Huanghe var* that were fed a diet containing 1 × 10^6^ CFU/g live *L. delbrueckii* showed higher SOD, CAT, GSH-Px, and T-AOC and lower MDA concentrations in an 8-week trial [49]. Similarly, Dawood et al. reported that dietary supplementation with heat-killed *L. plantarum* at a concentration of 100 mg/kg feed significantly increased SOD and CAT activities and decreased MDA levels in the serum in a 12-week trial on farmed tilapia (*Oreochromis niloticus*) [48]. Previous studies have shown that *L. casei* K17 demonstrates strong scavenging activity against hydroxyl radicals, 2,2-diphenyl-1-picrylhydrazyl, and superoxide anions both in vitro and vivo [16]. Consistent with these studies, when compared with SG, our study indicated that LB significantly increased SOD, GSH-Px, and T-AOC activities in serum and GSH-Px activity in the liver, while decreasing liver MDA activity. MB significantly increased SOD, GSH-Px, and T-AOC activities in serum and SOD activity in the liver, while decreasing liver MDA activity. DB significantly increased SOD activity in serum and GSH-Px and SOD activities in the liver. Furthermore, results demonstrated that LB, DB, and MB enhanced serum and liver antioxidant capacity in *M. salmoides*.

The liver is important for metabolizing products from the digestive tract, and histological changes are considered a good indicator for evaluating nutritional conditions [50]. Morphometric parameters are most used as indicators of the metabolic activity of hepatocytes. These parameters include hepatocyte number, surface area, and nuclear area, along with glycogen and lipid content of the cytoplasm [51]. Zhao et al. showed that *L. casei* dietary supplementation in a basal diet supplemented with 6 × 10^6^ CFU/g for 31 days could alleviate piglet lipopolysaccharide-induced liver injury by reducing proinflammatory cytokines and increasing antioxidative capacity [52]. In this study, *M. salmoides* that were fed the MB and LB diets showed regular hepatocyte morphology with cytoplasmic lipid vacuoles that left hepatocyte size unaltered. However, higher occurrence rates of hepatocyte swelling, hepatocyte vacuolization, nucleus deviation, and cellular periphery cytoplasmic vacuolization were observed in the hepatocytes of fish fed with SG, MG, and FS diets; the structures of liver sinusoids were not obvious in *M. salmoides* fed the same diets. The increase in hepatocellular vacuolization observed in the SG, MG, and FS groups was due to the accumulation of very large lipid droplets, which reflects the higher lipid content in *M. salmoides* liver in comparison to *M. salmoides* that were fed MB or LB diets. Previous studies have indicated that alterations in the size and volume of hepatocytes may be due to a physiological response to excess lipid and energy storage [53]. This confirmed that dietary supplementation with LB and MB could maintain normal liver histology and preserve liver function. TBA is a sensitive indicator of acute liver cell damage and can be used to detect liver cirrhosis and liver damage. In this study, decreased TBA levels in the SG group indicated that oxidative stress induced hepatic dysfunction and hepatotoxicity in *M. salmoides*, but dietary LB, DB, and, especially, MB reduced damage to *M. salmoides* liver cells (Figure 3 and Figure 6).

Dietary administration of probiotics, leading to reduced TG, TC, and/or LDL-C levels and increased HDL-C levels, has been shown to induce a hypolipidemic effect in fish [15,29]. Yang et al. reported that supplementation with 10^9^ CFU/kg *B. cereus* significantly improved TC, LDL-C, and HDL-C levels in Pengze crucian carp serum [15]. Cao et al. also reported, through a 70-day feeding trial on Pengze crucian carp, that supplementation with 1 g/kg β-glucan and 1 × 10^9^ CFU/kg *B. subtilis* acted as a hypolipidemic by decreasing TC and LDL-C levels and increasing HDL-C levels in serum [14]. Meanwhile, Ye et al. reported, in a 56-day trial, that administration of 10^7^ cells/g *B. clausii* significantly decreased TG and LDL-C levels in the Japanese flounder (*Paralichthys olivaceus*) [29]. In this study, DB, LB, and MB were found to improve lipid metabolism by increasing the effectiveness of HDL-C and reducing TC, TG, and LDL-C levels, indicating that dietary addition of 10^8^ CFU/g DB, LB, and MB had a hypolipidemic effect in *M. salmoides*. Insufficient fermentation broth dose may be a reason for the FS diet not showing any significant difference in LDL-C and TC levels when compared with the SG diet. Meanwhile, both LB and MB diets showed a consistent trend in serum and liver samples, possibly because the beneficial substances produced by *L. casei* K17 in the fish body were absorbed to stimulate SOD and GSH-Px activity or lead to the expression or inhibition of related genes to further stimulate the body’s SOD and GSH-Px activities, thus enhancing antioxidant capacity. Using *L. casei* K17 as an additive for *M. salmoides* feed could accelerate lipid peroxidation and reduce fat accumulation in the liver. Furthermore, improved antioxidant capacity could reduce free radicals and other products produced during lipid peroxidation, which damaged cells. Specifically, the number of nuclear offset cells and the degree of adipocyte vacuolization decreased, as observed through liver cell morphology.

## 5. Conclusions

The dietary supplementation of *M. salmoides* with 10^8^ CFU/g LB, MB, and DB increased immunity and antioxidant levels. Furthermore, and more importantly, these diets improved lipid metabolism, maintained normal liver histology, and preserved liver function in *M. salmoides* after a trial for 69 days. LB and MB diets also promoted *M. salmoides* fillet quality.

## Figures and Tables

**Table 1:** Different processing methods for *M. salmoides* feed.

Treatment Group	Feed
Saline group (SG)	300 g basic feed + 30 mL 0.85% sterile saline
Milk group (MG)	300 g basic feed + 30 mL 10% skim milk powder
Live bacteria (LB)	300 g basic feed + 30 mL live *L. casei* K17 10^8^ CFU/mL
Live bacteria protected by skim milk powder (MB)	300 g basic feed+ 30 mL (10% skim milk powder + live *L. casei* K17 10^8^ CFU/mL)
Dead bacteria (DB)	300 g basic feed + 30 mL dead *L. casei* K17 10^8^ CFU/mL
Fermentation supernatant (FS)	300 g basic feed + 30 mL fermentation supernatant

**Table 2:** The association between fillet texture and final individual weight of *M. salmoides* based on the analysis of covariance.

Group		SG	MG	LB	MB	DB	FS
Hardness (g)	Unadjusted	490.67 ± 147.03	524.67 ± 96.55	729.33 ± 119.49	945.33 ± 264.13 ^b^	590.00 ± 95.14	718.00 ± 151.25
	Adjusted	456.05 ^a^ ± 109.37	499.00 ^a^ ± 102.27	739.66 ^a^ ± 94.44	972.02 ^a^ ± 102.99	622.51 ^a^ ± 107.55	708.76 ^a^ ± 94.13
Stickiness (g.s)	Unadjusted	5.81 ± 0.81	7.37 ± 1.70	7.81 ± 2.45	9.22 ± 2.36	7.46 ± 3.09	7.97 ± 3.55
	Adjusted	6.11 ^a^ ± 1.76	7.59 ^a^ ± 1.65	7.72 ^a^ ± 1.52	8.99 ^a^ ± 1.66	7.18 ^a^ ± 1.73	8.05 ^a^ ± 1.52
Springiness (mm)	Unadjusted	0.517 ± 0.068	0.457 ± 0.012	0.437 ± 0.061	0.483 ± 0.058	0.437 ± 0.061	0.487 ± 0.035
	Adjusted	0.541 ^a^ ± 0.035	0.475 ^a^ ± 0.033	0.429 ^a^ ± 0.030	0.464 ^a^ ± 0.033	0.413 ^a^ ± 0.034	0.493 ^a^ ± 0.030
Chewiness (mJ)	Unadjusted	109.96 ± 33.80	120.74 ± 21.92	207.34 ± 11.04	252.05 ± 40.90 ^b^	184.54 ± 30.05	170.69 ± 24.92
	Adjusted	112.01 ^a^ ± 20.35	122.26 ^a^ ± 19.03	206.73 ^a^ ± 17.57	250.48 ^a^ ± 19.16 ^b^	182.62 ^a^ ± 20.01	171.24 ^a^ ± 17.51
Gumminess (g)	Unadjusted	315.12 ± 126.06	345.54 ± 90.57	489.50 ± 82.82	609.30 ± 128.59 ^b^	374.02 ± 55.57	442.65 ± 109.35
	Adjusted	304.47 ^a^ ± 72.26	337.64 ^a^ ± 67.56	492.676 ^a^ ± 62.39	617.51 ^a^ ± 68.04	384.02 ^a^ ± 71.06	439.81 ^a^ ± 62.19
Cohesiveness	Unadjusted	0.65 ± 0.05	0.64 ± 0.06	0.65 ± 0.06	0.61 ± 0.03	0.63 ± 0.07	0.67 ± 0.03
	Adjusted	0.68 ^a^ ± 0.03	0.67 ^a^ ± 0.03	0.64 ^a^ ± 0.03	0.59 ^a^ ± 0.03	0.60 ^a^ ± 0.03	0.68 ^a^ ± 0.03

Unadjusted values (mean ± SD, *n* = 9), Adjusted values (mean ± SE, *n* = 9), SD Standard deviation, SE Standard error, ^a^ Calculated in the analysis of covariance after adjusting for final weight, ^b^ significantly different from SG, *p* < 0.05.

## Data Availability

The data presented in this study are available in this paper.

## Supplementary Materials

The following are available online at https://www.mdpi.com/article/10.3390/ani11092564/s1, Figure S1: Sampling sites of fish for fillet quality. (A) Fillet composition. (B) Fillet texture.
