# Supplementary figures and images for: Dietary Lactobacillus casei K17 Improves Lipid Metabolism, Antioxidant Response, and Fillet Quality of Micropterus salmoides
Source: Animals (Basel). 2021 Aug 31;11(9):2564. doi: 10.3390/ani11092564 (PMC8471337; doi:10.3390/ani11092564)

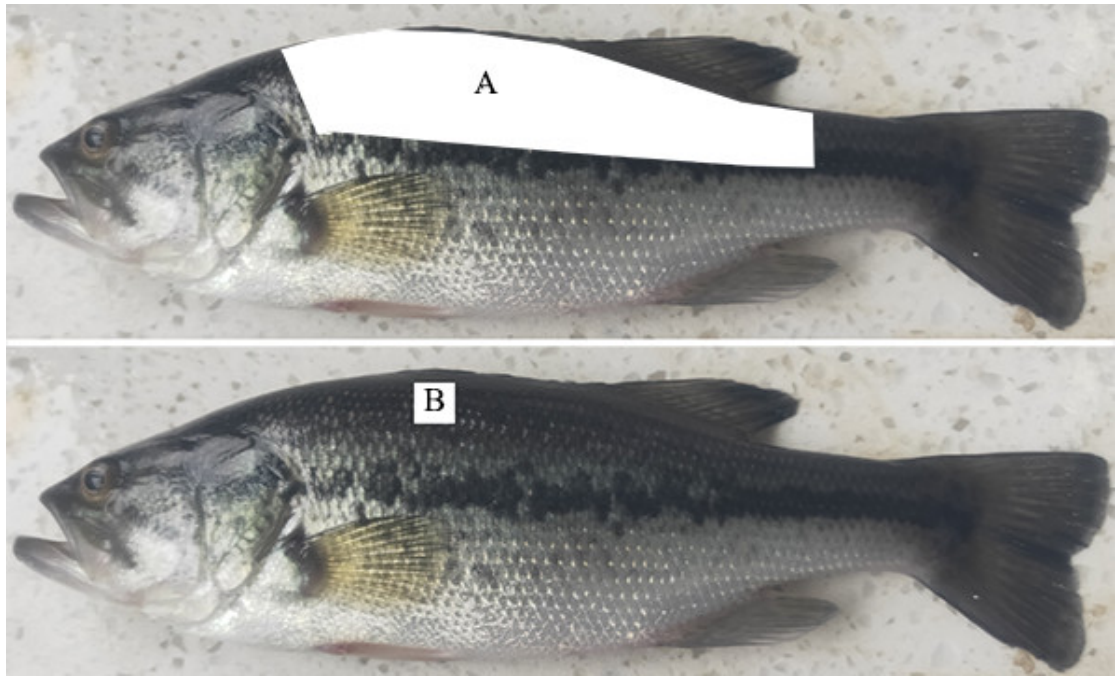

**Figure S1.** Sampling sites of fish for fillet quality. **(A)** Fillet composition. **(B)** Fillet texture.

Supplement: Supplementary file 1 [file animals-11-02564-s001.zip › animals-1285444-supplementary.pdf]
